# Evaluation of geoimputation strategies in a large case study

**DOI:** 10.1186/s12942-018-0151-y

**Published:** 2018-07-31

**Authors:** Naci Dilekli, Amanda E. Janitz, Janis E. Campbell, Kirsten M. de Beurs

**Affiliations:** 1 Center for Spatial Analysis, University of Oklahoma, 3100 Monitor Ave. Suite 180, Norman, OK USA; 2 The University of Oklahoma Health Sciences Center, 801 NE 13th Street, Oklahoma City, OK USA; 3 Department of Geography and Environmental Sustainability, University of Oklahoma, 100 East Boyd Street, Norman, OK USA

**Keywords:** Geo-imputation, Address data, Coarse resolution, Census data, Demographics

## Abstract

**Background:**

Health data usually has missing or incomplete location information, which impacts the quality of research. Geoimputation methods are used by health professionals to increase the spatial resolution of address information for more accurate analyses. The objective of this study was to evaluate geo-imputation methods with respect to the demographic and spatial characteristics of the data.

**Methods:**

We evaluated four geoimputation methods for increasing spatial resolution of records with known locational information at a coarse level. In order to test and rigorously evaluate two stochastic and two deterministic strategies, we used the Texas Sex Offender registry database with over 50,000 records with known demographic and coordinate information. We reduced the spatial resolution of each record to a census block group and attempted to recover coordinate information using the four strategies. We rigorously evaluated the results in terms of the error distance between the original coordinates and recovered coordinates by studying the results by demographic sub groups and the characteristics of the underlying geography.

**Results:**

We observed that in estimating the actual location of a case, the weighted mean method is the most superior for each demographic group followed by the maximum imputation centroid, the random point in matching sub-geographies and the random point in all sub-geographies methods. Higher accuracies were observed for minority populations because minorities tend to cluster in certain neighborhoods, which makes it easier to impute their location. Results are greatly affected by the population density of the underlying geographies. We observed high accuracies in high population density areas, which often exist within smaller census blocks, which makes the search space smaller. Similarly, mapping geoimputation accuracies in a spatially explicit manner reveals that metropolitan areas yield higher accuracy results.

**Conclusions:**

Based on gains in standard error, reduction in mean error and validation results, we conclude that characteristics of the estimated records such as the demographic profile and population density information provide a measure of certainty of geographic imputation.

**Electronic supplementary material:**

The online version of this article (10.1186/s12942-018-0151-y) contains supplementary material, which is available to authorized users.

## Background

Spatial epidemiology is the study of geographic variation of diseases. Locational accuracy is essential in geographical studies including epidemiological studies where the locational characteristics and behaviors of the patient are key to understand the underlying risk factors to inform policymaking. For example, underlying spatial factors such as environmental exposures have been linked to cancer including, asbestos exposure and mesothelioma, polychlorinated biphenyls (PCBs) and melanoma, aflatoxin and liver cancer, benzene and acute myeloid leukemia, tobacco and multiple cancers, and air pollution and lung cancer [1–8]. Besides cancer, environmental exposures are also associated with other diseases, for example, air pollution has been linked with respiratory disease, cardiovascular disease, and reproductive health [9–12]. Spatial epidemiology and geographic information systems (GIS) have also been applied to non-environmental health issues, including understanding the built environment [13], health planning [14], and crime data [15].

While high quality scholarly research requires reliable locational information of exposures and outcomes, the level of spatial detail available to health researchers is often not sufficiently fine resolution. This is for two main reasons: (a) while health information is exceedingly valuable, it is protected by federal law and thus it is imperative to protect the privacy of individuals; and (b) information is often only collected or made available at a lower resolution, such as the zip code or county level. For health data, the exact geographic coordinates of participants are often not available without a data request and several levels of approval to ensure the confidentiality of participants is maintained. Failure to accurately and precisely capture geographic information may lead to incorrect findings and conclusions, including an overestimation of the true association [16] or imprecise estimates [17, 18]. Researchers often work with data with coarse resolution (e.g. when the complete street address is missing and only the ZIP code is available), resulting in omitted records and potentially creating biases due to misclassification [19]. Geocoding to ZIP code area centroids, a common practice in health research, often falsely indicates clustering at the centroid [20, 21], especially so in rural communities [22].

In order to rectify issues associated with imprecise spatial data, several spatially informed geo-imputation methods have been developed to increase the spatial resolution. They are similar to disaggregation methods [15], which interpolate data at smaller units using the spatial distribution of ancillary data.

Geo-imputation strategies can generally be divided into stochastic and deterministic methods. One method for stochastic geo-imputation is the use of the cumulative distribution function to randomly assign a case to a locale [23–25]. A variety of this method uses a variable such as population to construct the probability of a locale being chosen [24]. Deterministic methods assign cases to locales deterministically, based on a set of rules, such as the geographically weighted mean of locales, or the centroid of the locale [26] that is the best fit. A mixture of the two can be used as well by selecting a random point within a deterministically chosen locale. Although the literature on the use of imputation for missing address information is sparse, authors have used both stochastic and deterministic methods, including alone and in combination [23, 27–32]. For example, Curriero et al. [23] found that misclassification in assignment of correct census tract was reduced most using deterministic geoimputation weighted by specific ethnicity/age population in comparison to a stochastic method and other types of deterministic methods. Walter and Rose [30] devised a stochastic method called random property allocation, which randomly assigns each case with incomplete address information to an address that was previously geocoded within the corresponding geographical unit, where each address has equal probability. They compared this method to one stochastic and three deterministic geo-imputation methods, which assign incomplete addresses to geographic centroids, population weighed centroids, areal proportion using random function and areal proportion using deterministic function, similar to the methods mentioned before. The authors observed that while all geo-imputation methods performed well, the random allocation method was the least prone to bias, as centroid based methods can create artificial clustering and bias.

The accuracy of geographic imputation methods is typically assessed by comparing the results from several methods. The results can be assessed based on: (a) the ratio of correct estimates, e.g. the number of times a case was assigned to the correct geographical unit; or (b) based on the distance between the predicted and known coordinates. Theoretically, when ground truth data is not available, it is not possible to evaluate the accuracy of the results. In this study, we are not focused on missing spatial data but rather our focus is on the application of geo-imputation methods to improve the spatial accuracy of spatial data by estimating higher resolution locations of events or persons based on known lower resolution spatial information (such as ZIP code) and supporting information (such as demographic characteristics). In this study, we apply four geo-imputation methods, including both stochastic and deterministic, to impute coordinate level information followed by an evaluation of the performance of the geo-imputation methods using the demographic sub groups and the characteristics of the underlying geography.

## Methods

### Data

As discussed in the introduction, the development and discussion of geo-imputation methods is most relevant for the analysis of spatial interactions in disease patterns. However, since most of the actual hospital records are justifiably protected by privacy laws, we instead have selected a subset of the Texas Sex Offender Registry as the population to include in our case study. This dataset provides address, age, gender and race information on all convicted sex offenders in Texas [33]. Of the 88,552 records that were acquired at the access date (August 28, 2017), 52,260 had known Texas address information that were previously geocoded to X,Y coordinates. Of these records, 52,229 had a known race, and only this subset of the data with known address and race information was used in the subsequent analyses. A breakdown of the data by race, age and gender is provided in Table 1, which shows the existing race groups in the registry and summarizes records into five age groups. These records were spatially joined to the Texas Census Block layer to add the census block information. Here are the characteristics of this data that are relevant to this project [33]:

**Table 1:** Demographic summary of the Texas Sex Offender Registry 2017 used in the study

	White			Asian			Black			Hispanic			Native American			All		
	Male	Female	Total	Male	Female	Total	Male	Female	Total	Male	Female	Total	Male	Female	Total	Male	Female	Total
Age < 20	814	39	853	16	1	17	389	12	401	769	12	781	2	–	2	1990	64	2054
20 ≤ Age < 50	14,948	695	15,643	168	3	171	7653	160	7813	9538	229	9767	21	–	21	32,328	1087	33,415
50 ≤ Age < 65	7729	139	7868	50	–	50	3103	22	3125	2775	20	2795	6	–	6	13,663	181	13,844
65 ≤ Age < 85	1960	11	1971	8	–	8	355	1	356	553	–	553	2	–	2	2878	12	2890
Age ≥ 85	13	–	13	–	–	–	5	–	5	6	–	6	–	–	–	24	–	24
All	25,464	884	26,348	242	4	246	11,505	195	11,700	13,641	261	13,902	31	–	31	50,883	1344	52,227

- The sex offender registration laws in Texas went into effect on September 1, 1991. Among other information, The Texas Sex Offender Registration Program requires offenders to submit full name, date of birth, sex, race, height, weight, eye color, hair color, social security number, driver’s license number, shoe size, home address or a detailed description of each geographical location at which the person resides or intends to reside.
- Adult sex offenders must register either for life or for 10 years depending on certain conditions.
- Registered offenders must report address changes.
- Sex offenders may be prohibited from living in child safety zones defined by laws and city ordinances, as well as campuses of higher education.

This information means the data is not collected at specified, regular intervals, but whenever there is a new entry and as soon as possible after an address change. Demographic data for the population includes age, gender and ethnicity information from 2010 Census Summary File 1 (SF1) [34] at census block level, which is the target resolution to assign our data. The SF1 data includes nine ethnic groups and 23 population ranges for both males and females, resulting in 414 possible demographic combinations. Additional file 1: Tables S1 and S2 list the complete list of race and age groups in the Census data.

This demographic data was merged and joined to the census block shapefile with NAD 1983 Texas Centric Mapping System Albers projection. There are 914,231 census blocks in the State of Texas.

### Study design

For all records with existing X, Y coordinates, we carry out the following steps:

- Obtain census block information using GIS.
- Obtain census block group (to reduce data quality for the purposes of validating imputation).
- Impute X, Y coordinates based on census blocks with census information for each method.
- Calculate error distance for each strategy.

Thus, we first reduce the data quality of each X, Y record by selecting its block group in order to make the data comparable to the more coarse spatial data quality (steps a and b). In the next step (c), we attempt to improve the spatial data quality by correctly assigning each X, Y record to the correct census block. In the last step (d) we evaluate our results by calculating the distance between the point assigned to the census block, and the original X, Y record.

### Geo-imputation strategies

The chosen geo-imputation methods were derived from the literature, in order to assign a non-geocoded record with census block group information to (a) a random point within the entire census block group; (b) a random point within the extents of matching blocks; (c) the centroid of census block with the highest weight; and (d) the weighted centroid of the matching census blocks. We chose the following methods which are either stochastic or deterministic methods identified from the literature [23, 28–30].

### Imputation Strategy #1 and #2

The following two imputation methods generate geoimputed results based on a complete random spatial function. These methods provide a basis to test the relative usefulness of the deterministic methods (3 and 4) that rely on the underlying demographic characteristics.

Strategy #1, random in Block Group, assigns the record to a random point within the entire block group as used in Henry and Boscoe [28]. Strategy #2, random in Matching blocks, randomly assigns a random point only within blocks that have matching demographic population to the individual record.

### Imputation Strategy #3

This method assigns the record to the centroid of the geographical unit with the highest calculated weight. The weight is determined as in Eq. 1 [23]. For example, in the case of a 71-year-old white female person, the weight of a particular block would be calculated as the following:

1 $$Block\;Weight = \frac{No.\;of\;White\;Female\;70 - 74\;in\;Block}{Total\;of\;White\;Female\;70 - 74\;in\;Block\;Group}$$

This method can be prone to generating artificial clusters [29], as all imputed coordinates from a particular geographical unit (which is chosen as it has the highest weight) will be identical.

### Imputation Strategy #4

This strategy assigns the record to the weighted centroid of the matching census blocks using the mean center of the available population similar to the approach used by Walter and Rose [30]. However, we matched demographics to target smaller population (as in Eq. 1) rather than the general population only. This strategy requires first calculating the centroid of each census block, and then calculating one final centroid based on their imputation weights calculated according to Eq. 1. In this approach, the weight of each block is calculated using one of the 414 combinations based on the case’s gender, age, and race within the block group to which it belongs. This method can be compared to the previous imputation strategy, as both methods make use of the block weights. The difference is that this method results in an estimation built by the entire set of candidate geographies based on how likely they are to contain a specific case. Also, unlike the previous imputation strategy, this strategy is not prone to artificial clustering since it is nearly impossible to generate the same weighted mean center. Two imputations would overlap only if they are in the same census block group with identical demographic characteristics.

### Evaluation of the accuracy of the imputation strategies

We evaluate the imputation strategies described above by comparing the original coordinates with geo-imputed coordinates using all records. We calculated the distance between the imputed location and the actual location (accuracy), stratified by age groups and ethnicities since certain demographic groups may cluster spatially, while others distribute more uniformly. Thus, high accuracy location data can be deduced when the target demographics are very particular and only exist in one or a few candidate geographies. We also evaluated the results by population density, as we expected the results to be more accurate when the underlying geographical unit is smaller.

We developed box and whisker plots to display the accuracy of each geo-imputation method and underlying factors, validated the stochastic methods using multiple imputation, assess the sensitivity of results based on the administrative boundary type, and map the geographic inaccuracy. We expected to observe differences in geographical patterns of imputation accuracy across space (e.g. particular neighborhoods, rural areas, etc.).

## Results

We geoimputed 96.7% of records (n = 50,494) by all four strategies; the other 1734 records could not be geoimputed by Strategies #3 and #4 as they did not have any matching population in the searched census block group. This is potentially due to the difference in dates of data collection (Texas Sexual Offender Registry date of offense vs. US Census data restricted to 2010, the date census data was collected) combined with addresses change, or inaccuracies in data collection. In addition, many factors could account for this small number of records that could not be imputed including known racial misclassification issues [35, 36].

### Accuracy by imputation strategy and demographic group

**Fig. 1:**
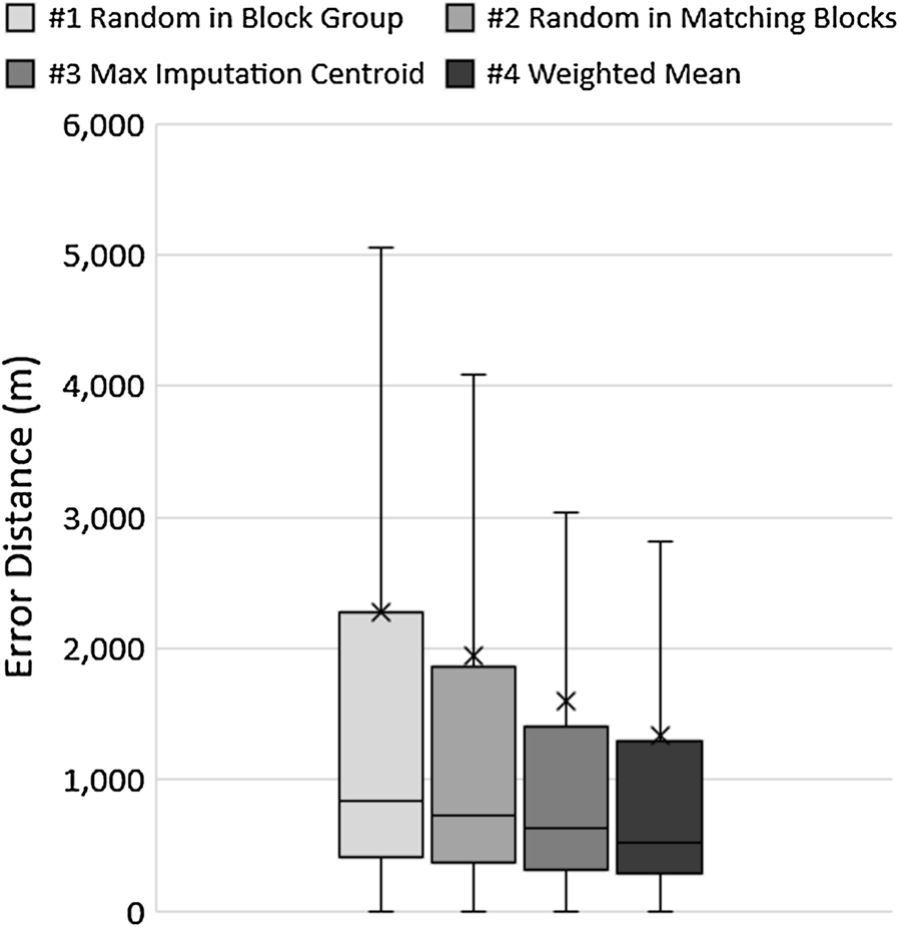
Box and Whisker plot of results by method (in meters)

Figure 1 displays the range of error distances (minimum, maximum, median, first quartile and third quartile) between each geoimputed location and the actual location in meters for the four imputation methods. We observed that the weighted mean method (Strategy #4) had the lowest error distance, followed by maximum imputation centroid (Strategy #3), random point in matching sub-geographies (Strategy #2) and random point in all sub-geographies (Strategy #1) methods. This indicates there is less uncertainty around the estimate of the mean measurement of the weighted mean method (Strategy #4), compared to random methods. In addition, the weighted mean method has a median error of 522 m, providing an almost 40% more accurate estimate than the complete random estimate method which revealed a median error of 845 m. Although the weighted mean method consistently reveals superior results, we observed differences among the demographic groups (Additional file 1: Table S3).

**Fig. 2:**
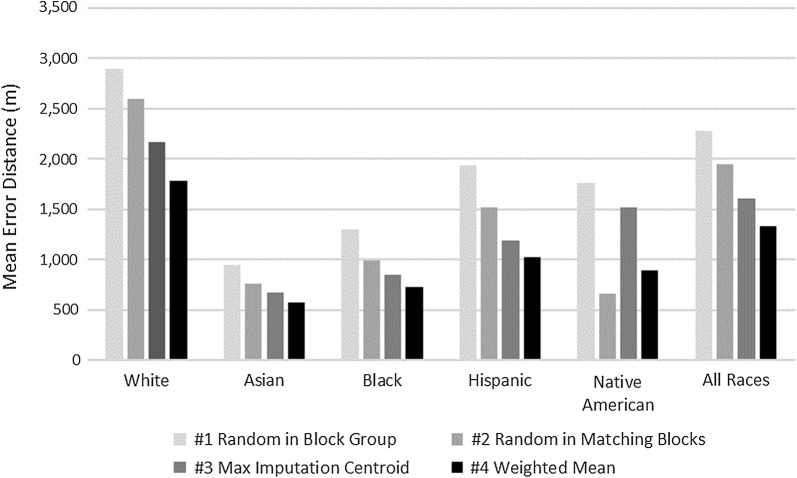
Mean error distance by race and method

**Fig. 3:**
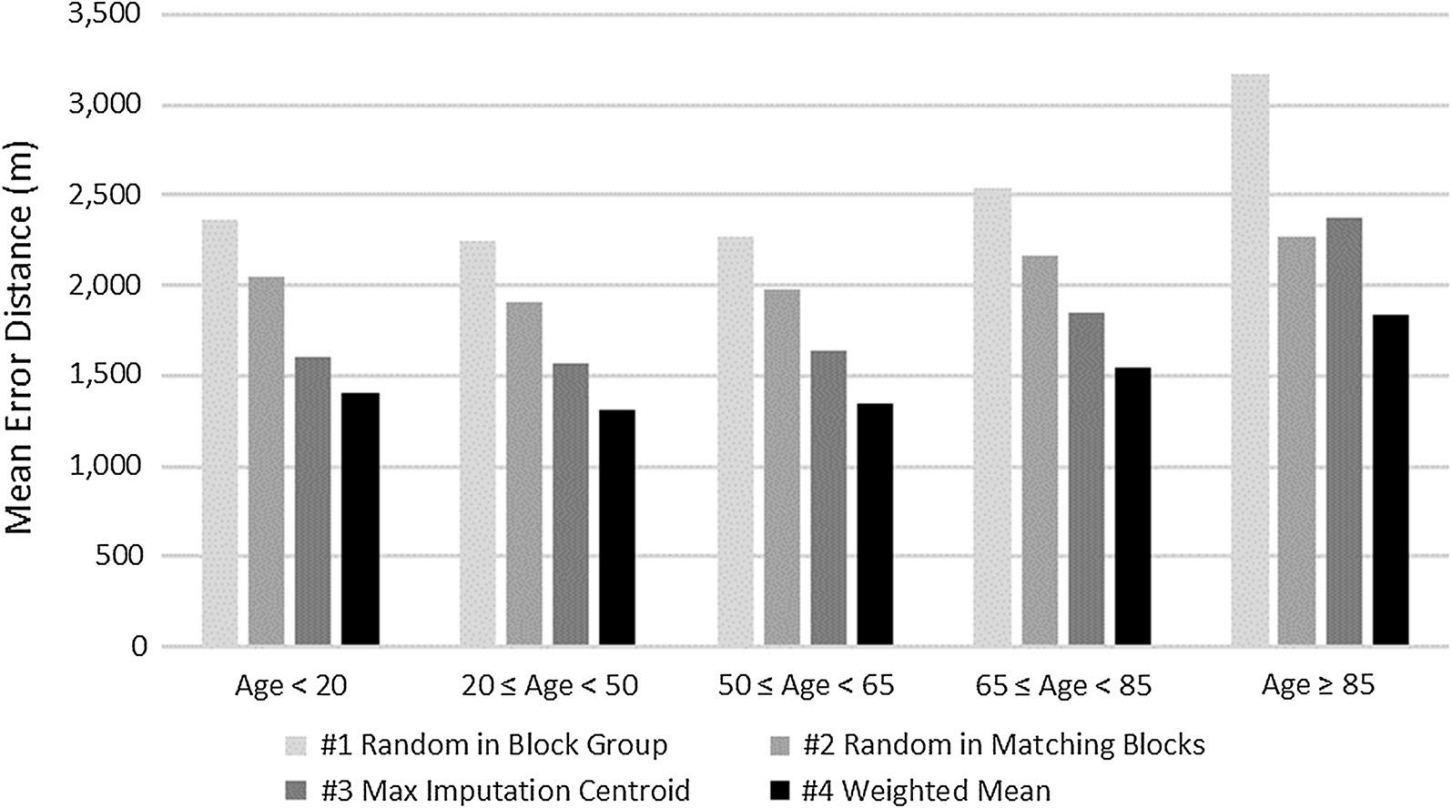
Mean error distance by age group and method

Accuracies were higher for minority populations because minorities tend to cluster in certain neighborhoods which makes it easier to impute their location (Fig. 2). However, the geoimputation accuracy appears to decline for older populations. This might be particularly due to elderly people living in rural areas (Fig. 3).

Figure 3 provides the mean error distance between the imputed points and the actual points broken down by age group and geo-imputation method (see Additional file 1: Table S3 for information on gender). These results also indicate that in estimating the actual location of a case, the weighted mean method (Strategy #4) is almost always the most superior for each demographic group followed by the maximum imputation centroid (Strategy #3), the random point in matching sub-geographies (Strategy #2) and the random point in all sub-geographies methods (Strategy #1). The only exception is the 65–85-year-old Asian subgroup, which has only 5 cases, as well as the American Indian/Alaska Native (AI/AN) group as a whole.

### Accuracy by imputation strategy and population density

**Fig. 4:**
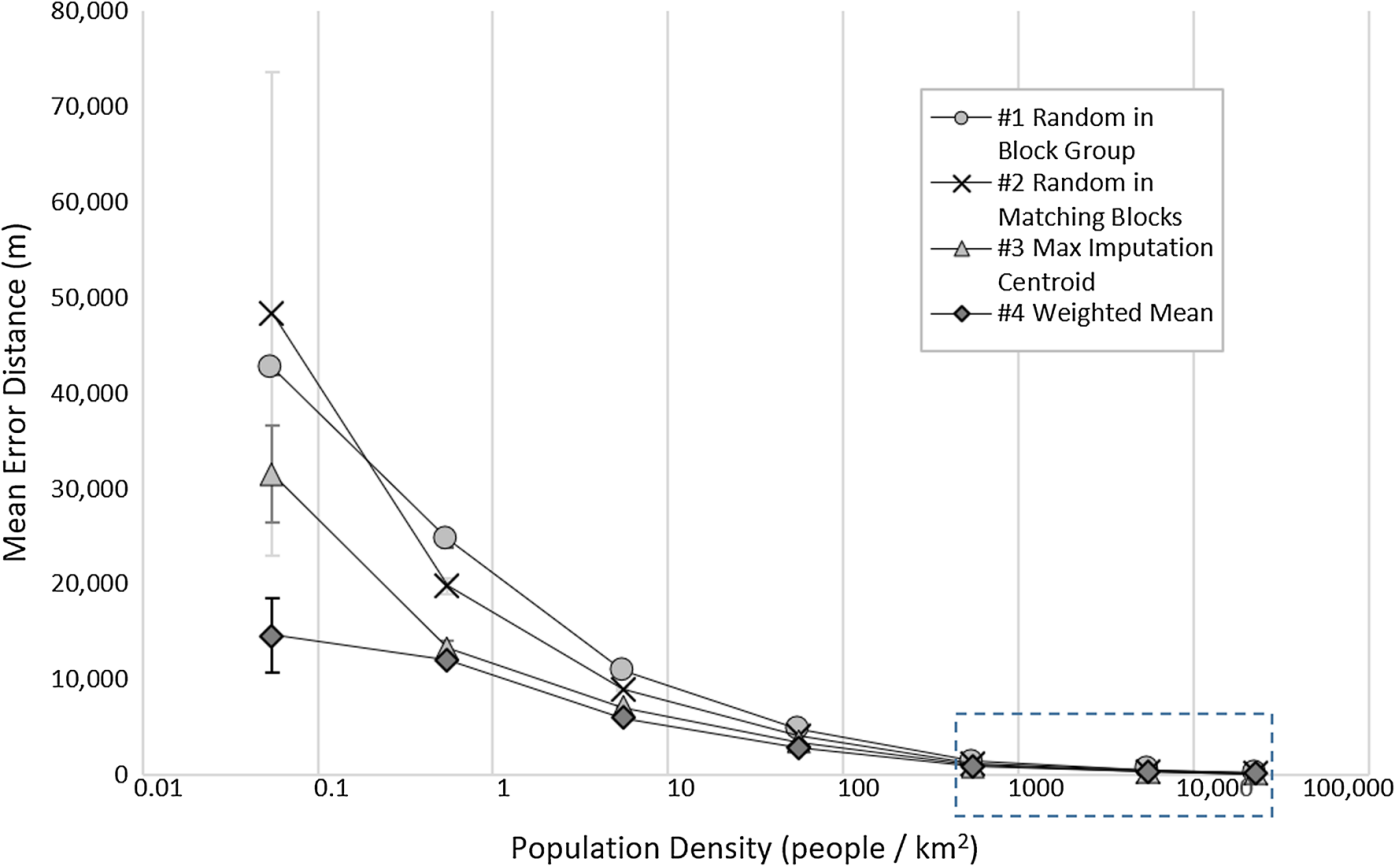
Mean error distance by population density and method

**Fig. 5:**
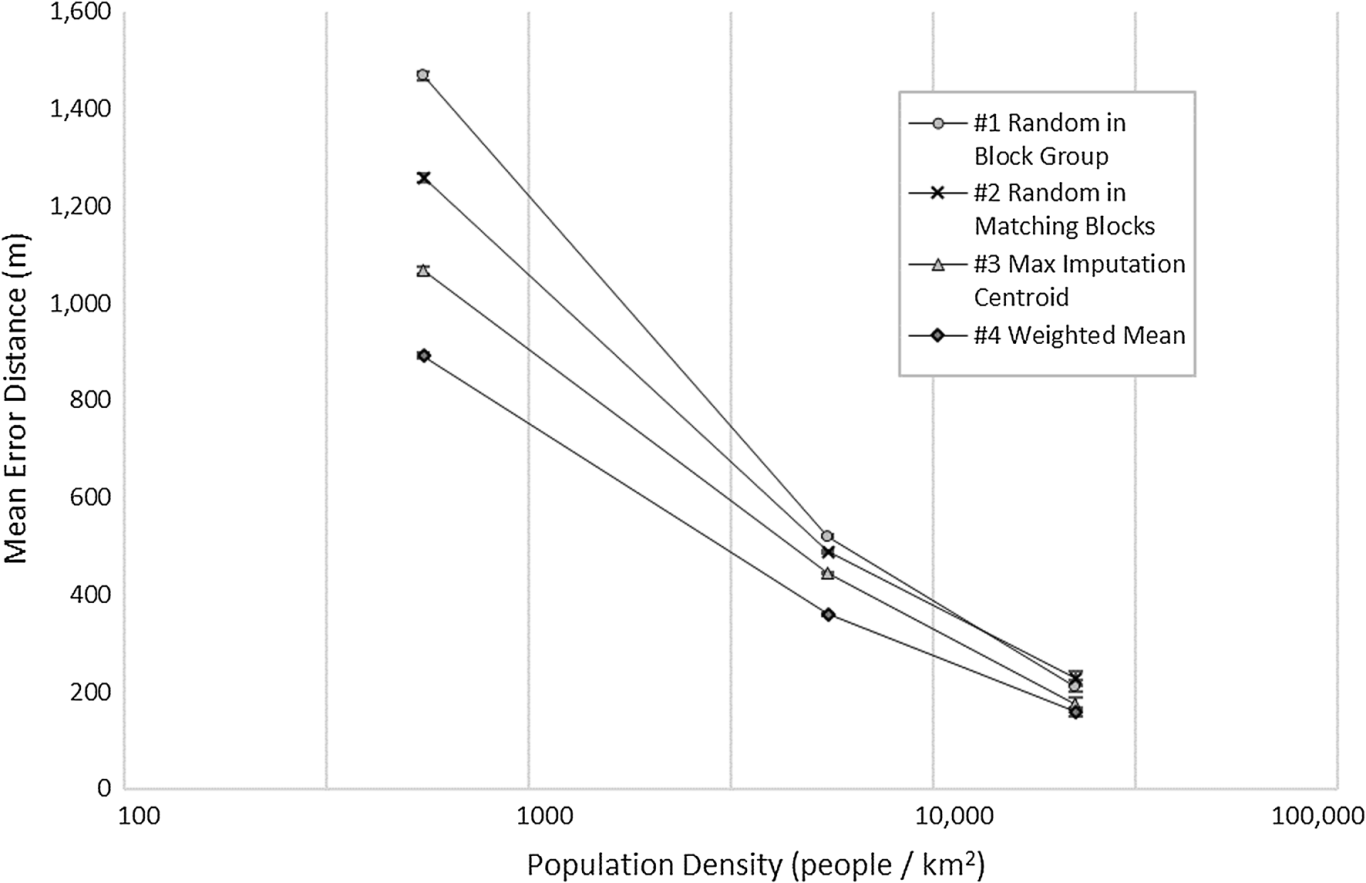
Mean error distance by population density and method in higher density areas

The population density varies greatly in the study area, from 0.08 people to 30,142 people per km^2^; thus, we applied a logarithmic scale to the x-axis to allow for the large differences in population densities. Figure 4 shows mean error distances as well as standard errors based on population densities. As is expected, all methods reveal a steep drop-off for increasing population densities with error distances and standard errors much larger in areas with very low population densities and lower error distances and standard errors for areas with higher population densities (for detailed information: Additional file 1: Table S4). For this reason, error bars are not visible at high density ranges. The weighted mean method reveals the lowest error distances for all population densities, which are indicative of the size of the geographical units. Figure 5 provides an enhanced view for areas with relatively high population densities (> 100 people/km^2^) only and indicates that the weighted mean method performs the best.

Certainly, it makes sense to expect high accuracies in areas with high population density, these areas are typically identified by smaller census blocks and decreasing the search space. Error results range from 48,300 m by a random estimate to only 58 m by the weighted mean estimate in our population density analysis. We observed that at the low end of the population density (0.01–0.1 people/km^2^), the performance of methods #1, #2, and #3 were not significantly different while method #4 performs significantly better than the first three methods. At the high end of the population density (10,000–35,000 people/km^2^) the methods fall into two groups with methods #3 and #4 performing better than methods #1 and #2 (Fig. 5).

### Multiple imputation

**Fig. 6:**
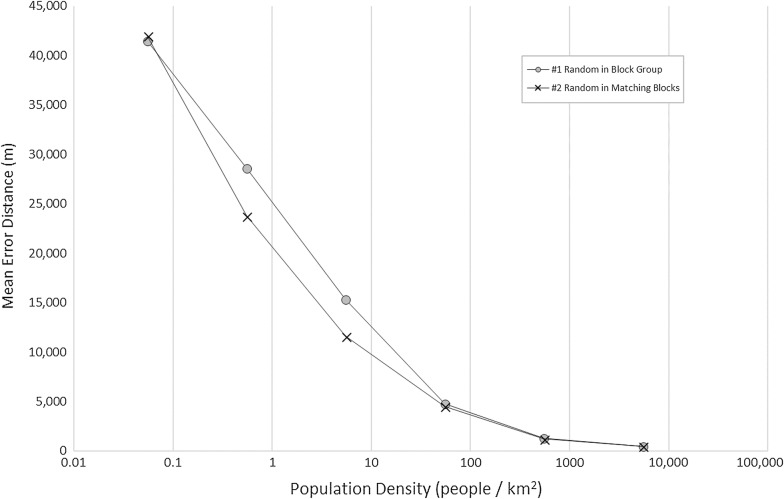
Mean error distance by population density using multiple imputation by random methods

We conducted multiple imputation using a sample size of 4864 records to further validate the results. We imputed each record 10 times and computed the error distance for each imputation for the methods #1 and #2, which are stochastic. We did not conduct multiple imputation for methods #3 and #4 since they are deterministic methods resulting in the same point estimate no matter how many times the method is run. We observed that the average error values from multiple imputation experience are comparable to the original results (Fig. 6).

### Sensitivity analysis

To evaluate the sensitivity with respect to different spatial units, we used the smaller sample set and reduced the spatial resolution first to census tracts and then to counties. We then conducted the identical analyses with all four strategies using these spatial units.

**Fig. 7:**
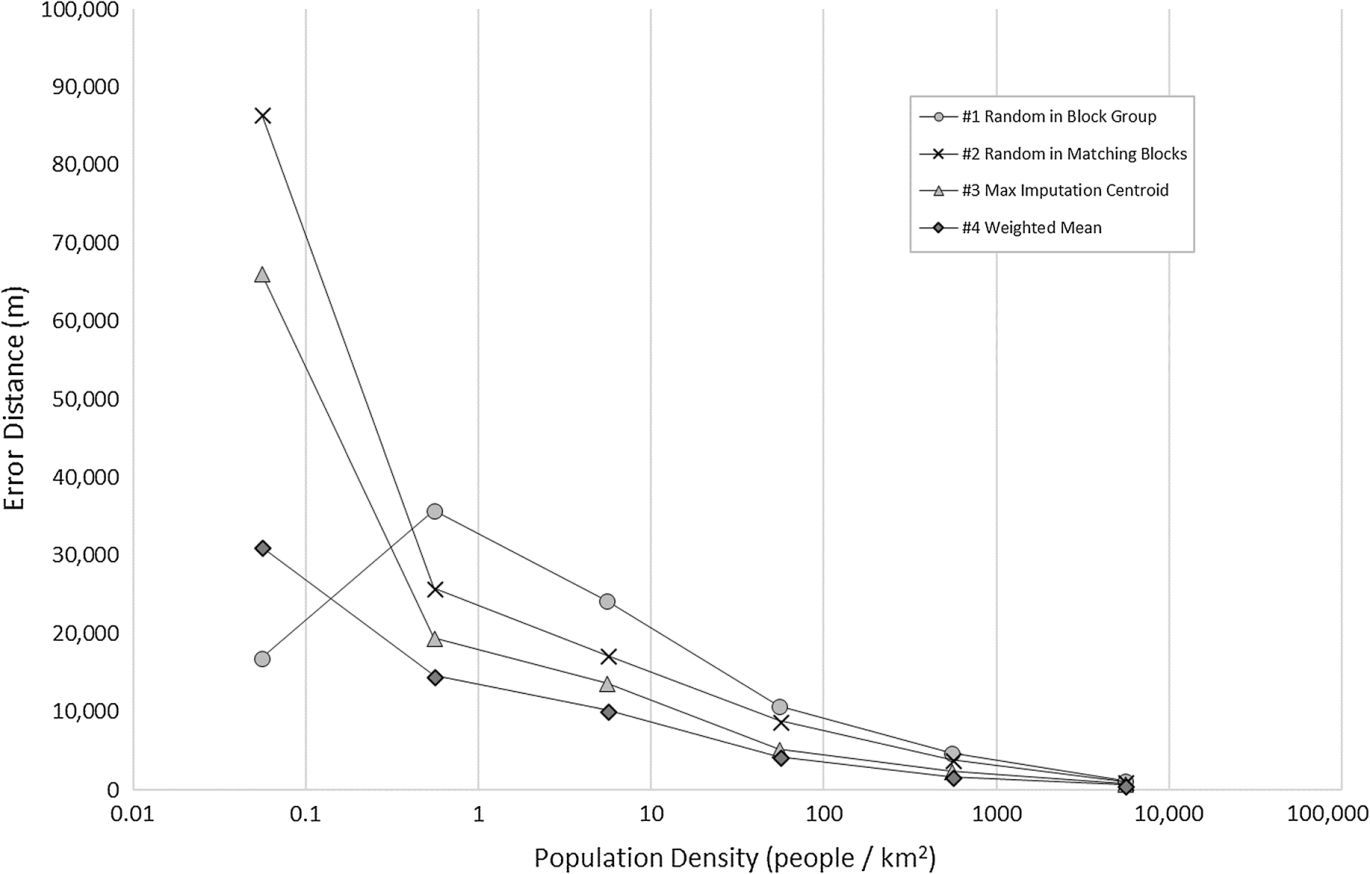
Mean error distance by population density using census tract level information

**Fig. 8:**
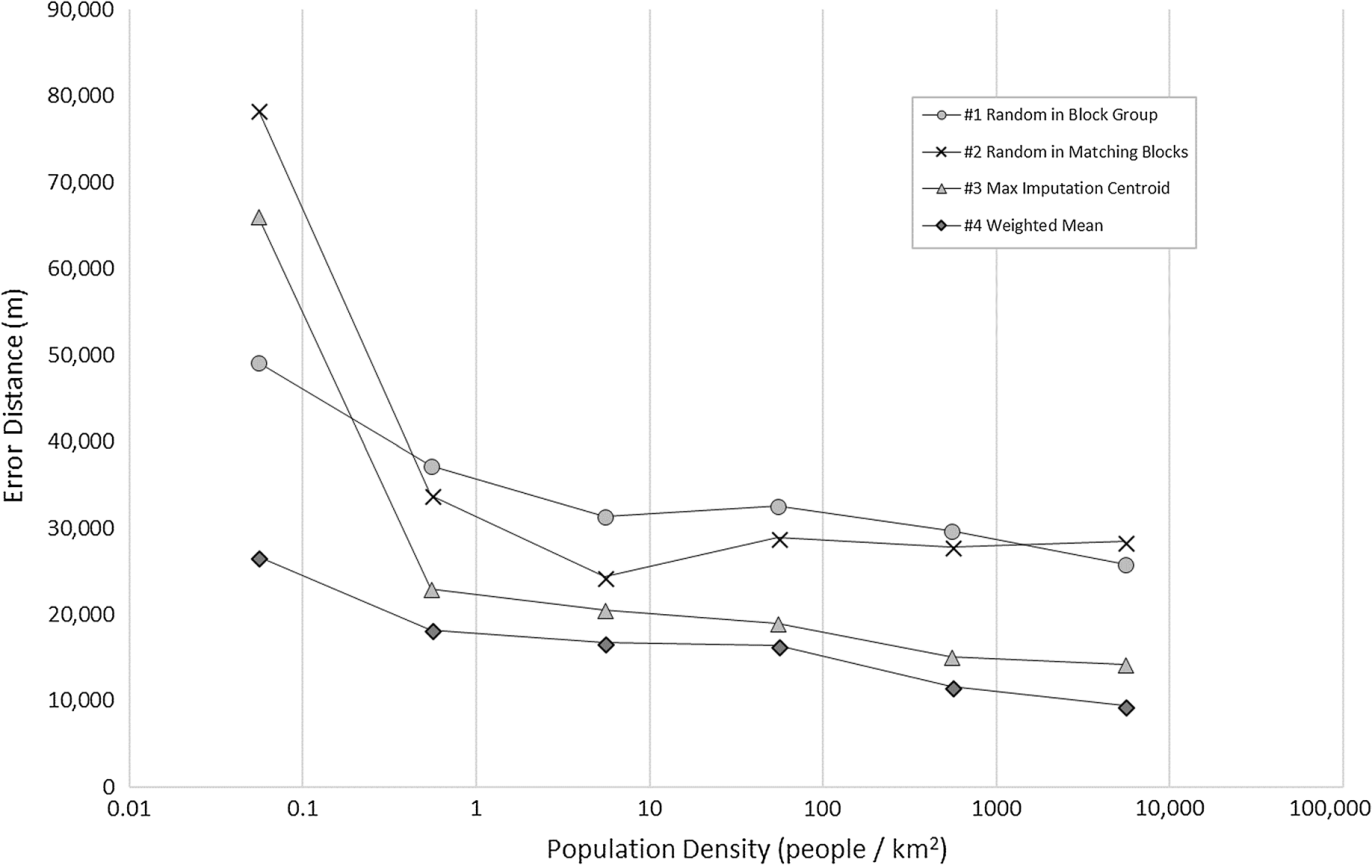
Mean error distance by population density using county level information

Figures 7 and 8 reveal that the difference between impacts of the census tract and county level information, respectively, depends on the underlying population density. There are only two imputations at the lowest density bin, which is the reason for the unexpected result for the first method. Overall, rural census tracts and counties have poorer performance compared to urban census tracts and counties.

### Accuracy across geography

**Fig. 9:**
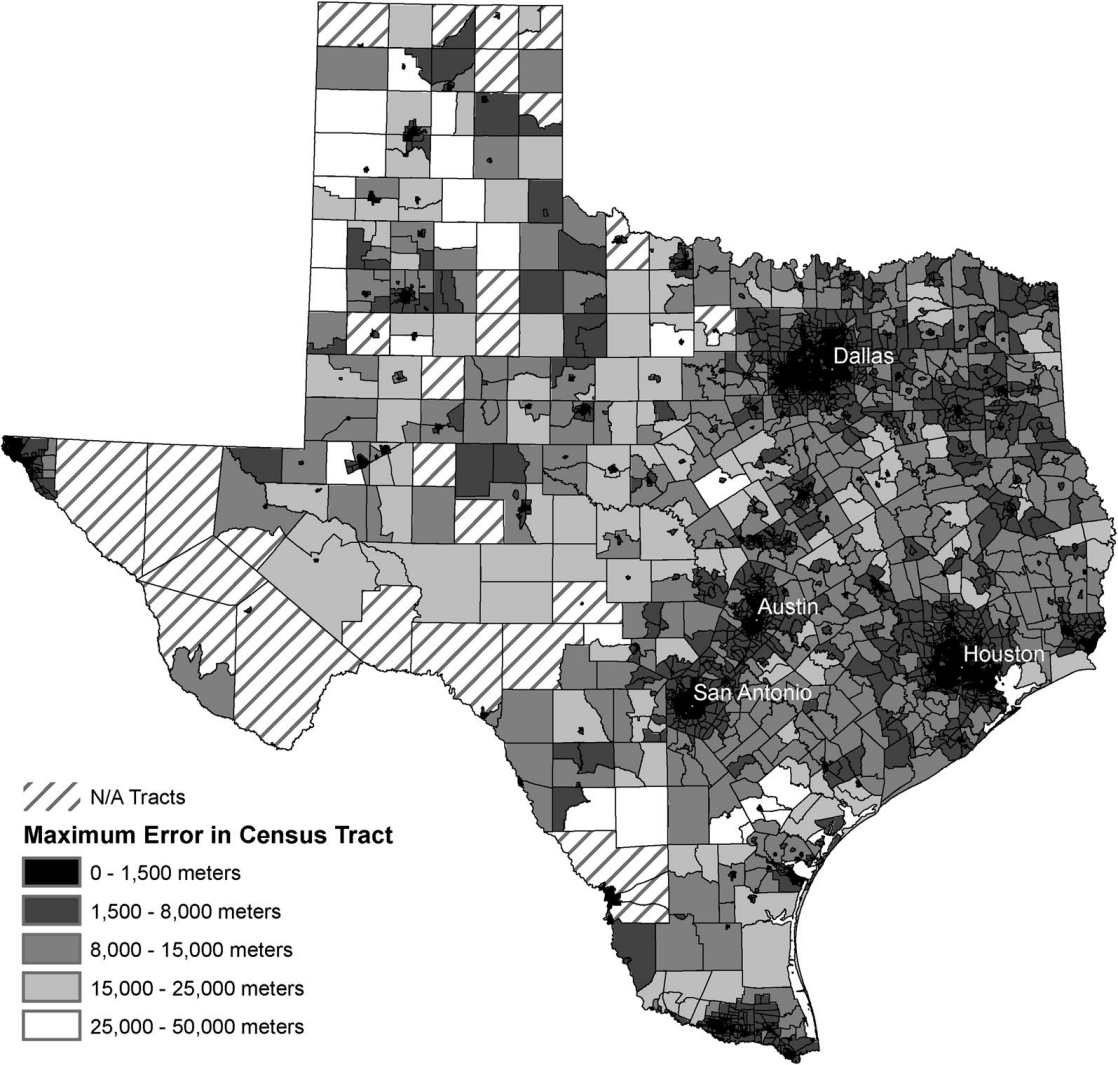
Interpolation of accuracy results of the weighted mean method

We plotted each of the 50,494 results on the map and performed an inverse distance weighted interpolation using weighted mean results (Fig. 9). Results, ranging from 52.8 to 47,992 m, reveal how the accuracy changes across the study area. As expected, Dallas, Houston, Austin and San Antonio metropolitan areas yield higher accuracy results. We aggregated the results to census tract level presenting the highest error distance and removed census tracts with < 0.001 imputed records per km^2^ to avoid reporting unreliable interpolation results.

## Discussions

We developed a methodological approach to evaluate various geoimputation methods with a large data set with complete, known, addresses and demographic information. Through rigorously evaluating the results stratified by demographic sub groups, population density, and geography, we have contributed additional knowledge to the field [23–25, 30].

This approach can be applied in contexts with missing addresses to increase the spatial resolution of the existing information. In such application, limitations and potential uncertainties of the geoimputation can be deduced from the size and population density of the underlying geography, as well as particular characteristics of the demographical profile of the particular record. We found that strategy #4, the Weighted Mean method, performed the best overall as in Curriero et al. [23], and in almost all sub evaluation criteria. This also supports Henry and Boscoe’s [24] stochastic method weighted by race and ethnicity population as opposed to a random point or a geographic centroid. As in Henry and Boscoe’s [24] study, we presented results by ethnicity, age and population density (also as in Walter and Rose [30] who evaluated them based on Metropolitan vs. Non-Metropolitan areas), but with further detail. Similar to Hibbert et al. [25], which report results by geography (i.e. four states), we report the results across the space. As in JD Hibbert et al. [36] and FC Curriero et al. [34], we also conducted multiple imputation of a sample set to validate our results. In addition, we conducted sensitivity analyses using two coarser spatial units of census tracts and counties. All the reviewed studies evaluated results based on assignment to correct unit, while we reported the results based on distance between predicted point and the actual point. We argue that correct assignment probability depends on the number of high resolution units in the search space, and therefore reporting the error distance can be viewed as an alternative way to evaluate different methods.

The range of error based on the demographic characteristics and population density is instructive for researchers working with limited locational information. For example, for some exposures of interest, sub kilometer gains in accuracy in urban core areas may not be very significant on epidemiological associations. On the other hand, the accuracy gains based on certain demographic groups or population densities (such as rural areas) may provide required level of accuracy to establish such associations.

There are a few potential issues in the evaluation of the results:

- We use demographic data from the Census Bureau from 2010. If the demographics of the case’s location changed from the time of the decennial census, our error estimates would be impacted. Similarly, the individuals on the sex offender registry could have moved, and would report a different address to the registry than the address reported at the census. This might explain some of the 1734 records that could not be geoimputed by Strategies #3 and #4.
- Potential data collection inaccuracies could also result in misclassification of race/ethnicity. For example, there are 13,336 records reporting a white race with unknown ethnicity, which we corresponded as white race in the Census data. We also corresponded the 26,135 records with white race with Hispanic ethnicity to the Hispanic or Latino race category in the Census data (Additional file 1: Table S1). While we assumed that other attributes are collected correctly, while there is no feasible way to validate them.
- While the sexual offender dataset is very large, it may still not be representative of the general population in terms of demographics as well as living preferences/limitations. For example, only 2.5% of the records belong to females. This is not comparable to many other health outcomes where these methods may be applied, including cancer rates among men and women, which ranges between 7:4 and 3:2 [37]. Additionally, we assume that the individuals in this database are randomly dispersed throughout the community, which is unlikely because of their status as a sexual offender.

Many publicly-available datasets, including state-level cancer incidence and mortality data and the Surveillance Epidemiology and End Results program data are generally available at the county level, prohibiting detailed analysis with complete address information due to privacy and confidentiality concerns. Although additional validation in other datasets that include both genders are needed, these methods are generalizable to other publicly-available datasets. Future studies may apply these methods to other types of health data with missing complete address information and to data sources that lack certain demographic information, at the expense of generating additional uncertainty.

Future studies should consider conducting geoimputation at other geographic levels, including ZIP code level information or applying a method based on cumulative random function, which would assign cases to finer spatial units randomly based on weights. Theoretically the deterministic imputation methods used in this study can be further improved by utilizing additional data, such as income, education etc., if available. These methods can be further enhanced by additional GIS or Remote Sensing data to exclude areas that do not contain residences. For example, we could eliminate areas without any residential buildings with a combination of GIS (zoning) and RS (impervious surface) data and methods.

## Conclusions

Based on gains in standard error, reduction in mean error and validation results, we conclude that methods #3 and #4, Maximum Imputation and Weighted Mean methods were preferable in this study when no fine level spatial information is available, though this should be replicated in a population that is more randomly dispersed. We conclude that characteristics of the estimated records such as the demographic profile and population density information impact accuracy of results. In the absence of ground truthing information, such variables can provide accuracy information using the error ranges provided in this study.

## Additional file

**Additional file 1.**
