# Supplementary material for: Evaluation of geoimputation strategies in a large case study
Source: Int J Health Geogr. 2018 Jul 31;17:30. doi: 10.1186/s12942-018-0151-y (PMC6069790; doi:10.1186/s12942-018-0151-y)
Supplement: Supplementary file 1 — Additional file 1. [file 12942_2018_151_MOESM1_ESM.docx]

**Additional File**

**Additional File: Table S1**

| **Code** | **Race** |
| --- | --- |
| A | White Alone |
| B | Black or African American Alone |
| C | American Indian and Alaska Native Alone |
| D | Asian Alone |
| E | Native Hawaiian and Other Pacific Islander Alone |
| F | Some Other Race Alone |
| G | Two or More Races |
| H | Hispanic or Latino |
| I | White Alone, Not Hispanic or Latino |

**Additional File: Table S2**

| **Age Group** |
| --- |
| Age < 5 |
| 5 ≤ Age ≤ 9 |
| 10 ≤ Age ≤ 14 |
| 15 ≤ Age ≤ 17 |
| 18 ≤ Age ≤ 19 |
| Age = 20 |
| Age = 21 |
| 22 ≤ Age ≤ 24 |
| 25 ≤ Age ≤ 29 |
| 30 ≤ Age ≤ 34 |
| 35 ≤ Age ≤ 39 |
| 40 ≤ Age ≤ 44 |
| 45 ≤ Age ≤ 49 |
| 50 ≤ Age ≤ 54 |
| 55 ≤ Age ≤ 59 |
| 60 ≤ Age ≤ 61 |
| 62 ≤ Age ≤ 64 |
| 65 ≤ Age ≤ 66 |
| 67 ≤ Age ≤ 69 |
| 70 ≤ Age ≤ 74 |
| 75 ≤ Age ≤ 79 |
| 80 ≤ Age ≤84 |
| 85 ≤ Age |

**Additional File: Table S3:** Mean Error Distance in Meters by Geo-Imputation Method and Demographic Profile

|  | **#1**  **Random in Block Group** | | | **#2**  **Random in Matching Blocks** | | | **#3**  **Max Imputation Centroid** | | | **#4**  **Weighted Mean** | | |
| --- | --- | --- | --- | --- | --- | --- | --- | --- | --- | --- | --- | --- |
| **White** | **Male** | **Female** | **All** | **Male** | **Female** | **All** | **Male** | **Female** | **All** | **Male** | **Female** | **All** |
| **Age < 20** | 3,255.8 | 2,477.8 | 3,219.9 | 2,839.1 | 2,274.7 | 2,813.1 | 2,268.4 | 2,348.7 | 2,272.1 | 1,976.7 | 1,918.5 | 1,974.0 |
| **20 ≤ Age < 50** | 2,934.0 | 2,975.4 | 2,935.8 | 2,617.5 | 2,754.0 | 2,623.5 | 2,184.9 | 2,165.5 | 2,184.1 | 1,793.3 | 1,761.7 | 1,791.9 |
| **50 ≤ Age < 65** | 2,794.0 | 2,233.9 | 2,784.2 | 2,538.1 | 2,151.7 | 2,531.4 | 2,108.5 | 1,712.5 | 2,101.6 | 1,726.6 | 1,403.5 | 1,721.0 |
| **65 ≤ Age < 85** | 2,849.4 | 2,139.3 | 2,846.3 | 2,579.2 | 2,127.0 | 2,577.3 | 2,224.2 | 1,642.2 | 2,221.7 | 1,827.0 | 1,381.6 | 1,825.0 |
| **Age ≥ 85** | 5,019.9 | - | 5,019.9 | 3,722.1 | - | 3,722.1 | 3,943.1 | - | 3,943.1 | 3,099.9 | - | 3,099.9 |
| **All** | 2,896.3 | 2,831.2 | 2,894.1 | 2,598.0 | 2,634.1 | 2,599.2 | 2,168.1 | 2,098.4 | 2,165.7 | 1,781.9 | 1,709.5 | 1,779.5 |
|  |  |  |  |  |  |  |  |  |  |  |  |  |
| **Asian** | **Male** | **Female** | **All** | **Male** | **Female** | **All** | **Male** | **Female** | **All** | **Male** | **Female** | **All** |
| **Age < 20** | 736.7 | 1,045.2 | 760.4 | 712.7 | 442.5 | 691.9 | 633.9 | 359.3 | 612.7 | 621.8 | 458.4 | 609.2 |
| **20 ≤ Age < 50** | 977.4 | 287.6 | 966.8 | 804.6 | 1,247.6 | 811.4 | 713.2 | 296.8 | 706.8 | 571.4 | 508.6 | 570.5 |
| **50 ≤ Age < 65** | 959.0 | - | 959.0 | 604.3 | - | 604.3 | 591.5 | - | 591.5 | 573.2 | - | 573.2 |
| **65 ≤ Age < 85** | 809.8 | - | 809.8 | 511.4 | - | 511.4 | 338.4 | - | 338.4 | 531.5 | - | 531.5 |
| **Age ≥ 85** | - | - | - | - | - | - | - | - | - | - | - | - |
| **All** | 953.1 | 540.1 | 946.2 | 753.1 | 979.3 | 756.8 | 674.8 | 317.6 | 668.9 | 574.0 | 491.9 | 572.7 |
|  |  |  |  |  |  |  |  |  |  |  |  |  |
| **Black** | **Male** | **Female** | **All** | **Male** | **Female** | **All** | **Male** | **Female** | **All** | **Male** | **Female** | **All** |
| **Age < 20** | 1,240.3 | 650.8 | 1,222.5 | 1,080.1 | 579.2 | 1,065.0 | 888.4 | 456.5 | 875.3 | 759.3 | 332.2 | 746.4 |
| **20 ≤ Age < 50** | 1,315.2 | 1,009.7 | 1,309.2 | 998.5 | 904.7 | 996.7 | 860.6 | 760.9 | 858.6 | 742.3 | 629.3 | 740.1 |
| **50 ≤ Age < 65** | 1,254.7 | 790.6 | 1,251.6 | 971.0 | 720.5 | 969.3 | 816.6 | 492.5 | 814.4 | 687.9 | 389.3 | 685.9 |
| **65 ≤ Age < 85** | 1,482.8 | 165.2 | 1,478.6 | 1,063.1 | 265.0 | 1,060.5 | 848.8 | 540.2 | 847.8 | 793.9 | 265.5 | 792.2 |
| **Age ≥ 85** | 694.4 | - | 694.4 | 359.5 | - | 359.5 | 226.1 | - | 226.1 | 165.6 | - | 165.6 |
| **All** | 1,300.9 | 957.8 | 1,295.4 | 995.3 | 860.1 | 993.1 | 849.0 | 710.4 | 846.8 | 729.4 | 581.7 | 727.0 |
|  |  |  |  |  |  |  |  |  |  |  |  |  |
| **Hispanic** | **Male** | **Female** | **All** | **Male** | **Female** | **All** | **Male** | **Female** | **All** | **Male** | **Female** | **All** |
| **Age < 20** | 2,058.7 | 1,374.4 | 2,048.2 | 1,752.5 | 1,032.3 | 1,741.4 | 1,284.8 | 857.0 | 1,278.2 | 1,133.6 | 555.2 | 1,124.7 |
| **20 ≤ Age < 50** | 1,896.9 | 2,272.4 | 1,905.8 | 1,490.2 | 1,650.0 | 1,494.0 | 1,172.2 | 1,187.1 | 1,172.6 | 998.7 | 1,121.9 | 1,001.6 |
| **50 ≤ Age < 65** | 1,987.0 | 2,690.4 | 1,991.8 | 1,576.3 | 2,531.5 | 1,582.9 | 1,245.5 | 2,090.2 | 1,251.3 | 1,049.2 | 1,596.6 | 1,053.0 |
| **65 ≤ Age < 85** | 2,086.9 | - | 2,086.9 | 1,372.8 | - | 1,372.8 | 1,134.1 | - | 1,134.1 | 1,040.7 | - | 1,040.7 |
| **Age ≥ 85** | 1,069.0 | - | 1,069.0 | 596.2 | - | 596.2 | 668.6 | - | 668.6 | 392.9 | - | 392.9 |
| **All** | 1,931.2 | 2,261.5 | 1,937.5 | 1,517.7 | 1,685.9 | 1,520.9 | 1,191.8 | 1,237.8 | 1,192.6 | 1,018.0 | 1,130.4 | 1,020.1 |
|  |  |  |  |  |  |  |  |  |  |  |  |  |
| **Native American** | **Male** | **Female** | **All** | **Male** | **Female** | **All** | **Male** | **Female** | **All** | **Male** | **Female** | **All** |
| **Age < 20** | - | - | - | - | - | - | - | - | - | - | - | - |
| **20 ≤ Age < 50** | 2,004.4 | - | 2,004.4 | 707.1 | - | 707.1 | 1,752.3 | - | 1,752.3 | 979.7 | - | 979.7 |
| **50 ≤ Age < 65** | 805.1 | - | 805.1 | 458.9 | - | 458.9 | 562.3 | - | 562.3 | 562.3 | - | 562.3 |
| **65 ≤ Age < 85** | - | - | - | - | - | - | - | - | - | - | - | - |
| **Age ≥ 85** | - | - | - | - | - | - | - | - | - | - | - | - |
| **All** | 1,764.5 | - | 1,764.5 | 657.4 | - | 657.4 | 1,514.3 | - | 1,514.3 | 896.2 | - | 896.2 |
|  |  |  |  |  |  |  |  |  |  |  |  |  |
| **All Races** | **Male** | **Female** | **All** | **Male** | **Female** | **All** | **Male** | **Female** | **All** | **Male** | **Female** | **All** |
| **Age < 20** | 2,381.6 | 1,907.8 | 2,366.8 | 2,059.7 | 1,694.5 | 2,048.3 | 1,604.1 | 1,681.4 | 1,606.5 | 1,401.2 | 1,340.3 | 1,399.3 |
| **20 ≤ Age < 50** | 2,238.7 | 2,546.3 | 2,248.7 | 1,895.3 | 2,255.9 | 1,907.0 | 1,567.2 | 1,755.4 | 1,573.3 | 1,305.5 | 1,464.0 | 1,310.6 |
| **50 ≤ Age < 65** | 2,276.9 | 2,115.8 | 2,274.8 | 1,982.4 | 2,026.5 | 1,982.9 | 1,636.0 | 1,611.8 | 1,635.7 | 1,350.3 | 1,306.3 | 1,349.8 |
| **65 ≤ Age < 85** | 2,538.1 | 1,920.0 | 2,536.0 | 2,164.2 | 1,920.1 | 2,163.3 | 1,848.5 | 1,519.8 | 1,847.4 | 1,551.3 | 1,257.6 | 1,550.3 |
| **Age ≥ 85** | 3,167.1 | - | 3,167.1 | 2,268.1 | - | 2,268.1 | 2,381.1 | - | 2,381.1 | 1,836.3 | - | 1,836.3 |
| **All** | 2,270.9 | 2,454.9 | 2,275.6 | 1,939.5 | 2,196.8 | 1,946.1 | 1,602.5 | 1,731.2 | 1,605.8 | 1,334.6 | 1,435.9 | 1,337.2 |

**Additional File: Table S4:** Mean Error Distance in Meters by Geo-Imputation Method and Population Density of Starting Census Block (Population Density calculated as People per 1,000 m^2^)

| **Method Population**  **Density per 1,000 m^2^** | **Random in Block Group** | **Random in Matching Blocks** | **Max Imputation Centroid** | **Weighted Mean** |
| --- | --- | --- | --- | --- |
| **0 ≤ Pop Density < 0.0001** | 42,582.1 | 48,300.7 | 31,555.4 | 14,673.3 |
| **0.0001 ≤ Pop Density < 0.0005** | 30,120.4 | 21,651.5 | 13,458.5 | 11,971.4 |
| **0.0005 ≤ Pop Density < 0.001** | 22,354.7 | 18,984.7 | 13,229.5 | 12,166.3 |
| **0.001 ≤ Pop Density < 0.05** | 7,483.8 | 6,317.0 | 5,127.7 | 4,283.5 |
| **0.05 ≤ Pop Density < 0.1** | 3,221.7 | 2,763.7 | 2,321.3 | 1,878.1 |
| **0.1 ≤ Pop Density < 0.2** | 2,265.0 | 1,917.2 | 1,619.3 | 1,309.1 |
| **0.2 ≤ Pop Density < 0.4** | 1,706.6 | 1,448.3 | 1,196.3 | 1,055.6 |
| **0.4 ≤ Pop Density < 0.6** | 1,212.4 | 1,025.9 | 890.4 | 723.4 |
| **0.6 ≤ Pop Density < 1** | 884.2 | 793.3 | 694.0 | 567.3 |
| **1 ≤ Pop Density < 2** | 603.0 | 557.7 | 500.7 | 406.1 |
| **3 ≤ Pop Density < 20** | 378.6 | 365.4 | 329.6 | 273.5 |
| **20 ≤ Pop Density < 35** | 124.7 | 97.3 | 117.8 | 58.0 |
